# Expression of Functional Sphingosine-1 Phosphate Receptor-1 Is Reduced by B Cell Receptor Signaling and Increased by Inhibition of PI3 Kinase δ but Not SYK or BTK in Chronic Lymphocytic Leukemia Cells

**DOI:** 10.4049/jimmunol.1402304

**Published:** 2015-01-28

**Authors:** Kathleen J. Till, Andrew R. Pettitt, Joseph R. Slupsky

**Affiliations:** Department of Molecular and Clinical Cancer Medicine, University of Liverpool, Liverpool L69 3GA, United Kingdom

## Abstract

BCR signaling pathway inhibitors such as ibrutinib, idelalisib, and fostamatinib (respective inhibitors of Bruton’s tyrosine kinase, PI3Kδ, and spleen tyrosine kinase) represent a significant therapeutic advance in B cell malignancies, including chronic lymphocytic leukemia (CLL). These drugs are distinctive in increasing blood lymphocytes while simultaneously shrinking enlarged lymph nodes, suggesting anatomical redistribution of CLL cells from lymph nodes into the blood. However, the mechanisms underlying this phenomenon are incompletely understood. In this study, we showed that the egress receptor, sphingosine-1-phosphate (S1P) receptor 1 (S1PR1), was expressed at low levels in normal germinal centers and CLL lymph nodes in vivo but became upregulated on normal B cells and, to a variable and lesser extent, CLL cells following in vitro incubation in S1P-free medium. Spontaneous recovery of S1PR1 expression on normal B and CLL cells was prevented by BCR cross-linking, whereas treatment of CLL cells with idelalisib increased S1PR1 expression and migration toward S1P, the greatest increase occurring in cases with unmutated IgH V region genes. Intriguingly, ibrutinib and fostamatinib had no effect on S1PR1 expression or function. Conversely, chemokine-induced migration, which requires integrin activation and is essential for the entry of lymphocytes into lymph nodes as well as their retention, was blocked by ibrutinib and fostamatinib, but not idelalisib. In summary, our results suggest that different BCR signaling inhibitors redistribute CLL cells from lymph nodes into the blood through distinct mechanisms: idelalisib actively promotes egress by upregulating S1PR1, whereas fostamatinib and ibrutinib may reduce CLL cell entry and retention by suppressing chemokine-induced integrin activation.

## Introduction

Chronic lymphocytic leukemia (CLL) is a malignancy of mature B cells that can follow either a progressive or an indolent clinical course. Studies of mutational status and gene usage of the IgH V region (IGHV) of the BCR on CLL cells have not only revealed a relationship between IGHV mutation and clinical course, but have also led to wide acceptance of a key role for BCR engagement in disease pathogenesis and clinical behavior (1). One manifestation of progressive disease in CLL is the development of lymphadenopathy, which results from entry of malignant cells into lymph nodes, where they receive signals for survival and proliferation. In a normal lymph node, transendothelial migration (TEM) of B cells from high endothelial venules into the interfollicular area is stimulated by the chemokine CCL21 and is dependent on cell adhesion mediated by the integrin α_L_β_2_ (Supplemental Fig. 1A) (2–5). Once inside the lymph node, B cells then migrate to the follicles in a CXCL13-dependent manner in search of Ag (6). Exit, or egress, of B cells from lymph nodes depends on migration toward sphingosine-1 phosphate (S1P)–rich tissues such as the blood and occurs when the S1P receptor-1 (S1PR1; S1P_1_) is upregulated (7–11). S1PR1 is not expressed by peripheral blood cells, as high levels of its ligand S1P cause receptor internalization. However, when lymphocytes enter the S1P-depleted lymph node environment, the receptor is upregulated and mediates lymphocyte egress (11). In T cells, this process is prevented by activation of the TCR, which results in downregulation of S1PR1 (11). Importantly, the transit time of normal lymphocytes through lymph nodes is determined by levels of S1PR1 on the cell surface. Thus, lymphocytes that enter the lymph nodes but do not encounter Ag rapidly upregulate S1PR1 and transit through the node without delay. In contrast, T cells that encounter Ag downregulate S1PR1 due to repression by TCR signaling can remain within the lymph node for much longer periods of time (8, 10). The regulation of S1PR1 expression on normal B cells is unclear. However, normal B cells that have been chronically stimulated through their BCR do not migrate toward S1P (12), suggesting that S1PR1 expression may be repressed by BCR signaling.

The development of lymphadenopathy in CLL implies either enhanced entry of the malignant cells into lymph nodes and/or their retention within the node (13). As is the case with normal B cells, entry of CLL cells into lymph nodes is also driven by CCL21 (Supplemental Fig. 1B) (14–16). However, unlike normal B cells, CLL cells additionally require expression and activation of the integrin α_4_β_1_ to undergo TEM (15, 16). Once inside lymph nodes, CLL cells may respond to CXCL13 because they express high levels of CXCR5 (17). However, the relevance of CXCL13/CXCR5-dependent migration is uncertain because the nodal architecture of CLL lymph nodes is effaced. Retention of CLL cells in lymph nodes may result from enhanced adhesion to extracellular matrices (18) or from reduced expression of S1PR1 (19).

Recently, new therapeutic agents have been developed that target kinases involved in the BCR signaling pathway. These include idelalisib (CAL-101; GS-1101), ibrutinib (PCI-32765), and fostamatinib (R406), which inhibit, respectively, PI3Kδ (20), Bruton’s tyrosine kinase (BTK) (21), and spleen tyrosine kinase (SYK), although fostamatinib has additional activity against some other kinases (22, 23). All of these kinase inhibitors induce a rapid lymphocytosis associated with a reduction in lymphadenopathy when given to patients with CLL (24, 25). This strongly implies that these kinase inhibitors produce a mobilizing effect by redistributing CLL cells from the lymph nodes into the blood. In the case of ibrutinib, this effect has been attributed to blockade of BCR- and chemokine-induced integrin activation, resulting in reduced adhesion of CLL cells to lymph node stroma (26). However, given that CLL cells are chronically stimulated in vivo through their BCR (27–29) and that Ag receptor stimulation prevents upregulation of S1PR1 expression on normal T cells in lymph nodes, another possible explanation for the mobilizing effect of BCR pathway inhibitors presents itself. Namely, we hypothesized that S1PR1 expression is downregulated on CLL cells as a result of chronic BCR signaling and that this contributes to their retention within lymph nodes. We further speculated that BCR inhibitors relieve this BCR-mediated repression of S1PR1 expression, resulting in the egress of CLL cells from affected lymph nodes. We reasoned that these effects should be most evident in those cases with unmutated IGHV in which BCR signaling is particularly active (30).

We tested this hypothesis by examining the effects of BCR stimulation and BCR signaling pathway inhibitors on CLL cells of defined IGHV mutational status cultured in the absence of S1P. In keeping with our predictions, idelalisib increased the expression of S1PR1 and induced migration toward S1P, the greatest effect being observed in IGHV-unmutated CLL cells. In contrast, fostamatinib and ibrutinib had no such effect but, unlike idelalisib, inhibited CCL21-induced migration. Together, our findings suggest that idelalisib induces CLL cell mobilization by actively promoting S1P-directed egress from lymph nodes, whereas fostamatinib and ibrutinib mobilize CLL cells by releasing adhesive interactions and blocking chemokine-directed entry into lymph nodes.

## Materials and Methods

### Patient samples

This study was performed using peripheral blood samples from 20 patients with CLL; clinical data are shown in Supplemental Table I. Normal B cells were obtained from healthy donors (*n* = 6). Lymph node tissue was obtained from five patients with CLL and four healthy donors. The IGHV gene usage and extent of somatic hypermutation were determined by comparison with the nearest germline counterpart sequence in the international ImMunoGeneTics (IMGT) information system with IMGT/V-QUEST. A cutoff of 2% IGHV mutation was used to separate cases into mutated CLL and unmutated CLL groups (31, 32). HUVEC were purified from umbilical veins. All samples were obtained with informed consent and with the approval of the Liverpool Research and Ethics Committee, Royal Liverpool and Broadgreen University Hospitals National Health Service Trust and the Research and Development Committee, Liverpool Women’s Hospital National Health Service Trust.

### Cell preparation and culture

Primary B lymphocytes (normal and CLL) were used in all the experiments described. Cells were isolated from peripheral blood and buffy coats by centrifugation over Lymphoprep (Axis-Shield, Oslo, Norway). Normal B cells were purified by negative selection using a B cell isolation kit (Miltenyi Biotec, Bisley, U.K.) (>98% CD19^+^). All cultures involving primary lymphocytes employed RPMI 1640 medium containing 1% fatty acid-free BSA (Sigma-Aldrich, Poole, U.K.); HUVEC were cultured in IMDM containing 20% FCS, whereas HS-5 and CD40L-transfected fibroblasts were cultured in DMEM containing 10% FCS. All culture media were supplemented with 2 mM l-glutamine, 100 U/ml penicillin, and 100 μg/ml streptomycin (Invitrogen, Paisley, Scotland). Ibrutinib, fostamatinib, and idelalisib were all used at 1 μM (Selleckchem). These drug concentrations were sufficient to maximally inhibit the respective target kinases following BCR ligation (data not shown); IC_50_ values and peak plasma concentrations are shown in Supplemental Table II. Goat anti-human IgM [F(ab)_2_ fragments (Jackson ImmunoResearch Laboratories)] was used at 20 μg/ml.

### Immunohistochemistry

Tissue staining for S1PR1 was performed using formalin-fixed, paraffin-embedded tissue sections mounted on glass slides. S1PR1 (Abcam, Cambridge, U.K.) and IgG1 isotypic control (R&D Systems, Abbingdon, U.K.) Abs were used at 4 μg/ml, whereas CD20 and CD23 were supplied ready to use (Dako, Cambridge, U.K.). As previously described (33), dewaxing of the sections and Ag retrieval were performed with EnVision FLEX target retrieval solution with the Dako PT-link module. Slides were stained with an autostainer using the EnVision FLEX convenience kit (Dako) and counterstained in Meyers’ hematoxylin (Sigma-Aldrich). Isotypic control staining is shown in Supplemental Fig. 2A.

### Flow cytometry

Cells were simultaneously stained with directly conjugated mAbs to S1PR1 (clone 218713; R&D Systems), CCR7, CD49d (α_4_ integrin), and CD19 (all from BD Biosciences, Oxford, U.K.) together with appropriate isotypic control Abs and analyzed by multicolor flow cytometry. The percentage and mean fluorescence intensity for S1PR1, CCR7, and CD49d were determined on CD19^+^ cells. In addition, viability of the cells after culture was assessed using propidium iodide. None of the drugs used decreased the viability of CLL cells following culture. Because cells were cultured in conditions to minimize cell death, viability was >75% in the majority of cases.

### Migration assays

HUVECs were grown to confluence on the inserts of Transwell plates (5 μm pore size; Corning, High Wycombe, U.K.). S1P (Sigma-Aldrich) or CCL21 (R&D Systems) was added to the bottom wells at concentrations (100 ng/ml and 1 μg/ml, respectively) shown to induce maximum migration (data not shown). CLL cells were added to the inserts, and the number of B cells that had migrated to the bottom wells was counted after 6-h incubation. The migration index (number of CD19^+^ cells transmigrating with chemokine divided by number of cells transmigrating in the absence of chemokine) was then calculated.

### Statistical analysis

The Mann–Whitney *U* test was used to analyze differences in continuous measurement data between treated and untreated samples. χ^2^ analysis was used to relate categorical in vitro responses to patient characteristics.

## Results

### S1PR1 is underexpressed in normal germinal centers and CLL lymph nodes

**FIGURE 1.**
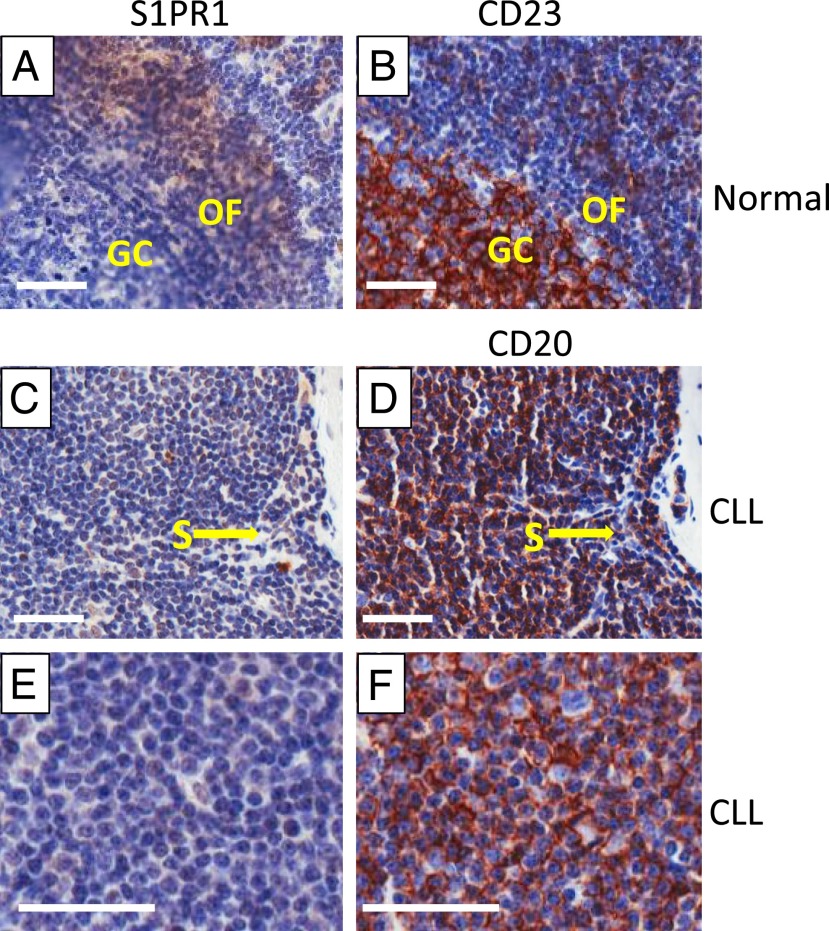
Expression of S1PR1 in normal and CLL node. (**A** and **B**) Normal lymph node. The cells of the germinal center (GC) are identified by staining for CD23, which is mainly expressed on activated B cells. Staining of the adjacent section for S1PR1 shows that CD23^+^ cells do not express S1PR1, whereas the cells within the outer follicle (OF) stain positively. (**C**–**F**) Sequential section of CLL lymph nodes stained for S1PR1 and CD20. In (C) and (D), it can be clearly seen that the endothelial cells lining the sinus (S) and other endothelial cells express S1PR1, whereas CD20^+^ CLL cells lack the receptor. (E) and (F) show a representative proliferation center in a CLL lymph node. CD20^+^ CLL cells do not express S1PR1. Parallel staining with the relevant isotypic control Ab is shown in Supplemental Fig. 2A. Scale bar, 50 μM; original magnification ×20.

Expression of S1PR1 within human lymphoid tissues has hitherto not been described. We therefore began our study by staining normal lymph nodes for S1PR1. As is shown in Fig. 1A and 1B, S1PR1 was expressed by all cells within the outer follicle, but not by CD23^+^ cells in the germinal center. In addition, the endothelial cells lining the sinus also stained for S1PR1, as did cells (presumably lymphocytes) within the sinus itself. We next examined lymph nodes from five different patients with CLL. In contrast to lymph nodes from healthy individuals, the majority of cells in the CD20^+^ CLL cells within the lymph nodes, including those in the proliferation centers, did not express S1PR1, although the sinus-lining endothelial cells were clearly positive (Fig. 1C–F). Thus, the pattern of expression of S1PR1 is as expected in normal lymph node tissue, but in CLL lymph nodes expression of this receptor appears to be downregulated.

### S1PR1 expression on normal B and CLL cells is downregulated by BCR signaling

**FIGURE 2.**
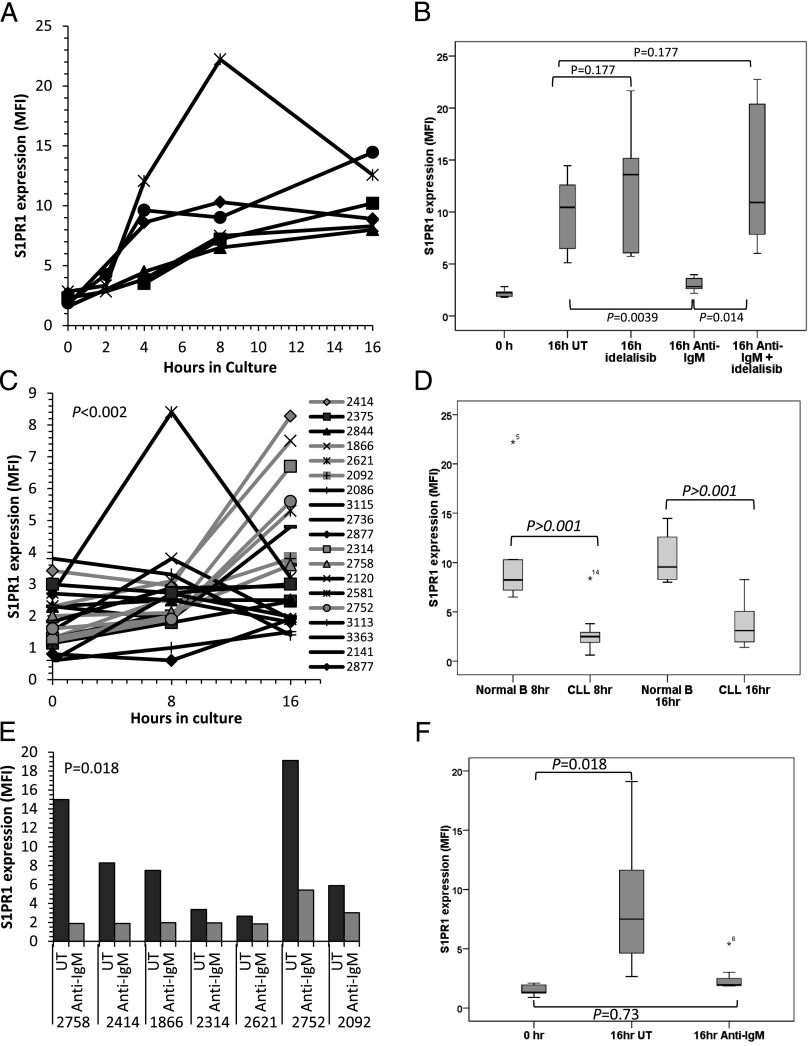
S1PR1 expression on normal and CLL B cells cultured in the absence of S1P. (**A**) Normal B cells from six healthy individuals were cultured for 16 h in medium lacking S1P and examined for S1PR1 expression by flow cytometry. An increase in S1PR1 was observed between 2 and 4 h and reached a peak at 8–16 h. (**B**) Normal B cells from six donors were cultured in S1P-free medium for 16 h in the presence or absence of anti-IgM and/or idelalisib (1 μM). S1PR1 expression was measured by flow cytometry. IgM cross-linking prevented the spontaneous increase in S1PR1 expression (*p* = 0.0039). Idelalisib had little effect on S1PR1 expression on normal B cells in the absence of IgM (*p* = 0.177); however, treatment reversed the anti-IgM–mediated suppression of S1PR1 expression (*p* = 0.014) with levels returning to those of untreated cells at 16 h (*p* = 0.177). (**C**) CLL cells from 20 patients were cultured for 16 h in S1P-free medium and examined for S1PR1 levels by flow cytometry. There was an overall increase in S1PR1 expression (*p* < 0.002), but the increase was variable, delayed, and generally of lower magnitude compared with that observed in normal B cells. (**D**) Comparison of S1PR1 upregulation in normal and CLL B cells at 8 and 16 h. The increase in expression was significantly greater in normal B cells at both time points. In the box-and-whiskers plot, the bar indicates the median of the mean fluorescence intensity values for S1PR1 expression, whereas an asterisk (*) identifies outlying data points that do not fall within the interquartile range. (**E**) Seven of the CLL samples showing most spontaneous increase in S1PR1 expression [highlighted in gray in (C)] were cultured in S1P-free medium in the presence or absence of anti-IgM and examined for S1PR1 expression by flow cytometry. Upregulation of S1PR1 was prevented by BCR cross-linking in all cases. (**F**) Pooled analysis of the seven cases of CLL described in (E) showing near-complete abrogation of spontaneous upregulation of S1PR1 expression (*p* = 0.73).

Most of the research describing S1PR1 expression and function in lymphocytes has been performed on mouse T cells. We therefore continued our characterization of this receptor on human cells by examining normal B cells cultured in the presence and absence of S1P, the presence of which is known to cause S1PR1 internalization (11). We found that S1PR1 expression spontaneously increased on normal B cells cultured in the absence of S1P (Fig. 2A, Supplemental Fig. 2B), an effect that is inhibited when S1P is present (Supplemental Fig. 2C). Consistent with the results of others studying the effects of Ag receptor engagement on S1PR1 expression in mouse T cells (11), we found that spontaneous upregulation of S1PR1 on human B cells cultured in the absence of S1P is similarly repressed by BCR cross-linking (Fig. 2B). Importantly, repression of S1PR1 expression by BCR cross-linking is reversed by pretreatment of normal B cells with idelalisib (Fig. 2B), suggesting a key role of PI3Kδ within the mechanism of this repression. Taken together, our culture and BCR stimulation experiments involving normal B cells are entirely in keeping with the observed distribution of S1PR1 expression in normal lymph node tissues (Fig. 1A), and support the notion that S1PR1 is internalized by its ligand in blood but re-expressed in the lymph node, where the levels of S1P are low unless it is downregulated by BCR signaling in the germinal center.

We next examined the effect of S1P withdrawal and BCR cross-linking on CLL cells. Similar to our findings in normal B cells, S1PR1 expression increased spontaneously on CLL cells when they were cultured in the absence of S1P (Fig. 2C). However, although the changes in S1PR1 expression reached statistical significance within the group of 20 cases tested (*p* < 0.002), the observed increase was delayed (no increase was seen at 4 h, and maximal levels were observed after 16 h) and variable, being of significantly smaller magnitude than the increase observed in normal B cells (*p* < 0.001; Fig. 2D). This variability could not be explained by IGVH mutational status because spontaneous upregulation of S1PR1 was similar in mutated and unmutated cases (*p* = 0.387; Supplemental Fig. 2D). Because the greatest increase in S1PR1 expression on CLL cells was seen at 16 h, we used this time point for all subsequent experiments.

We next sought to establish whether spontaneous S1PR1 upregulation expression was repressed by BCR signaling in CLL cells. To do this, the seven samples displaying the greatest spontaneous upregulation of S1PR1 at 16 h were cultured in the presence or absence of anti-IgM. In keeping with our observations in normal B cells, the spontaneous increase in S1PR1 was prevented in all seven cases of CLL by BCR cross-linking (*p* = 0.018; Fig. 2E, 2F). Collectively, these findings strongly support a role for BCR signaling in repressing the spontaneous upregulation of S1PR1 that is observed in the absence of S1P in both normal and CLL B cells.

### S1PR1 expression on CLL cells is increased by idelalisib but not fostamatinib or ibrutinib

**FIGURE 3.**
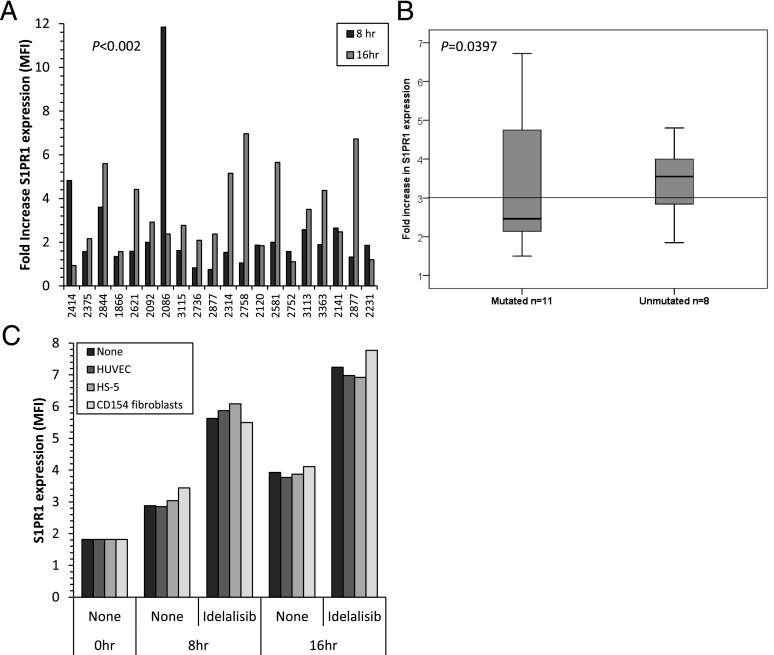
Effect of idelalisib on S1PR1 expression in CLL cells. (**A**) CLL cells from 20 cases were cultured in the presence or absence of idelalisib (1 μM) and examined by flow cytometry for S1PR1 levels. The chart shows the fold increase in mean fluorescence intensity compared with cells cultured for the same amount of time in the absence of idelalisib. The increase in S1PR1 expression was statistically significant at both time points (*p* < 0.002). (**B**) Box-and-whiskers plot of idelalisib-induced upregulation of S1PR1 at 16 h. Idelalisib induced an increase in S1PR1 expression in both IGHV-mutated (*n* = 11) and unmutated (*n* = 8) CLL samples, but the effect was greater in the latter cases (*p* = 0.0397). The bar represents the grand median of all samples. (**C**) CLL cells displaying little or no spontaneous recovery of S1PR1 expression (*n* = 4) were cultured in the presence or absence of idelalisib on different cell monolayers and examined by flow cytometry for S1PR1 levels. A representative example (patient 2141) is shown (data from individual cases are shown in Supplemental Fig. 2H). Idelalisib (1 μM) produced a marked increase in S1PR1 expression at 8 h irrespective of whether the CLL cells were cultured on endothelial cells (HUVEC), stromal cells (HS-5), or CD154-expressing fibroblasts.

We next addressed the question of whether BCR signaling could modulate S1PR1 expression in cases of CLL that displayed little or no spontaneous upregulation of S1PR1 during cell culture. We reasoned that failure of S1PR1 to upregulate in these cases most likely resulted from repression by constitutive BCR signaling. To test this idea, cells from the 20 CLL cases shown in Fig. 2C were cultured in S1P-free medium in the absence or presence of idelalisib at a concentration (1 μM) known to specifically inhibit PI3Kδ (34). In keeping with our hypothesis, we found that treatment of CLL cells with idelalisib resulted in a significant enhancement of spontaneous S1PR1 expression (Fig. 3A, Supplemental Fig. 2E). This result was in keeping with our observations in BCR-stimulated normal B cells, where idelalisib restored spontaneous expression of S1PR1 (Fig. 2B). The magnitude of S1PR1 upregulation induced by idelalisib varied between CLL cases, as did the time required for the maximum increase in S1PR1 expression to be observed. Nevertheless, the increase reached overall statistical significance at both 8 h (*p* = 0.002) and 16 h (*p* < 0.0001). In contrast to spontaneous upregulation of S1PR1, upregulation induced by idelalisib correlated with IGVH status, being higher in cases with unmutated IGHV genes (Fig. 3B; *p* = 0.0397). Importantly, inhibitors of other isoforms of PI3K (PI3Kα [A66] and PI3Kβ [TGX-221]) used at the same concentration (1 μM) did not promote spontaneous S1PR1 expression in any of the cases examined (Supplemental Fig. 2F; *p* > 0.14), indicating that the effect was isoform specific. Similarly, when we compared the effects of idelalisib with those of either fostamatinib or ibrutinib (all at 1 μM), we found that treatment of CLL cells with either of the latter two compounds had no effect on spontaneous S1PR1 expression (Supplemental Fig. 2G, 2H, respectively). Finally, coculture of CLL cells on endothelial cells (HUVEC), stromal cells (HS-5), or CD40L-expressing fibroblasts to mimic cell interactions in the lymph node microenvironment showed that none of these accessory cells had any effect on the expression of S1PR1 or its upregulation by idelalisib, nor did they render CLL cells responsive to S1PR1 modulation by fostamatinib or ibrutinib (Fig. 3C, Supplemental Fig. 2G–I). Taken together, these results indicate a specific role for PI3Kδ in the in vivo regulation of S1PR1 in both normal and CLL B cells that have been stimulated via the BCR.

### Migration of CLL cells toward S1P is enhanced by idelalisib, but not fostamatinib or ibrutinib

**FIGURE 4.**
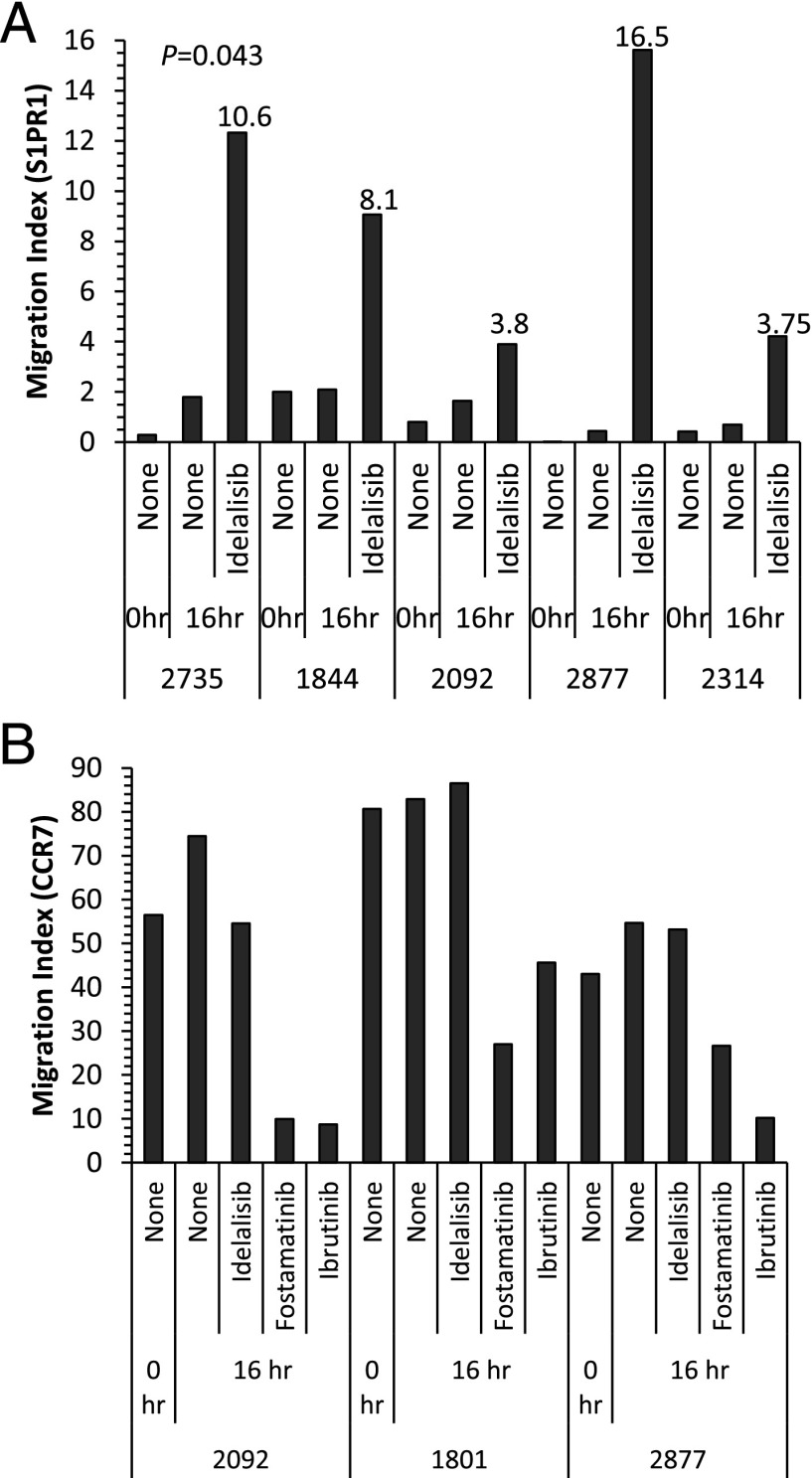
Effect of BCR inhibitors on TEM toward S1P and CCL21. (**A**) CLL cells from five patients were incubated for 16 h in the presence or absence of idelalisib (1 μM) and then examined for migration toward S1P using HUVEC-coated transwells. The numbers above bars indicate mean fluorescence intensity values for S1PR1 staining at the end of the incubation period. Idelalisib increased TEM toward S1P in all cases, the amount of migration correlating with levels of S1PR1. Untreated CLL cells underwent little or no migration. (**B**) CLL cells from three patients were cultured in the absence or presence of idelalisib, fostamatinib, or ibrutinib (all at 1 μM) and examined for migration toward CCL21 using HUVEC-coated transwells. Untreated CLL cells underwent TEM toward CCL21. Migration was reduced by fostamatinib and ibrutinib, but not idelalisib.

Having shown that idelalisib increases S1PR1 expression, we next sought to examine its effect on S1PR1 function. To do this, we examined the effect of the three BCR signaling inhibitors on CLL cell TEM toward S1P using a transwell system. As is shown in Fig. 4A, untreated CLL cells did not migrate toward S1P. When CLL cells were treated with idelalisib for 16 h before the assay, they displayed marked TEM toward S1P (*p* = 0.043), the amount of TEM observed correlating with the level of S1PR1 expression at the end of the preincubation period. In keeping with the inability of ibrutinib or fostamatinib to upregulate S1PR1, preincubation of CLL cells with either of these drugs did not enhance migration toward S1P (Supplemental Fig. 3A). These results demonstrate that the S1PR1 induced by idelalisib is functional and suggest that the CLL cell–mobilizing effect of idelalisib observed in vivo results at least in part from enhanced S1PR1-dependent egress of CLL cells from lymph nodes.

### Migration of CLL cells toward CCL21 is inhibited by ibrutinib and fostamatinib, but not idelalisib

Having elucidated the differential effects of idelalisib and fostamatinib/ibrutinib on S1PR1 expression and function, we next investigated the effects of these BCR signaling inhibitors on the processes responsible for entry of CLL cells into lymph nodes. Our previous work has shown that the latter process is dependent on α_4_β_1_ and CCL21 (15). We therefore examined the effect of the three inhibitors on CLL cell TEM toward CCL21. In keeping with the findings of other groups (35, 36), migration was blocked by preincubation of CLL cells with either fostamatinib or ibrutinib (Fig. 4B). This inhibitory effect did not result from altered expression of either α_4_β_1_ or CCR7 because no such change was observed (Supplemental Fig. 3B, 3C). In contrast to fostamatinib and ibrutinib, idelalisib had no effect on migration toward CCL21. Taken together with the experiments examining the effects of the three BCR signaling inhibitors on S1P migration, it can be deduced that the CLL cell–mobilizing effects of these drugs are mediated by different mechanisms; fostamatinib and ibrutinib inhibit signals required for entry and retention of CLL cells into lymph nodes, whereas idelalisib promotes egress by facilitating the upregulation of functional S1PR1.

## Discussion

The aim of this study was to elucidate the possible role of impaired egress as a determinant of lymphadenopathy in CLL, and relief of impaired egress as an explanation for the mobilizing effect of BCR signaling pathway inhibitors. We hypothesized that retention of CLL cells in the lymph nodes results at least in part from an inability to exit due to repression of S1PR1 expression by chronic BCR signaling, and that the CLL cell–mobilizing effects of BCR signaling inhibitors result from the reversal of such repression. To test this hypothesis, we first sought to confirm previous reports that S1PR1 expression is reduced in CLL as compared with normal B cells (19, 37, 38), and in addition demonstrate that S1PR1 expression is regulated by BCR signaling. To do this, we cultured the normal B and CLL cells in the absence of S1P to prevent receptor internalization and showed that spontaneous upregulation of S1PR1 could be prevented by BCR stimulation. These results are in keeping with a recent report that demonstrated that after long-term culture S1PR1 expression by CLL cells was downregulated by factors present in the microenvironment, including BCR signaling (37). For those cases of CLL that displayed little or no spontaneous recovery of S1PR1 expression during cell culture, we adopted a complementary approach involving treatment with BCR signaling inhibitors to block presumed endogenous BCR signaling. We tested the effects of three different BCR signaling inhibitors, all of which are in clinical development and have a potent CLL cell–mobilizing activity. We provide evidence that the mechanisms through which these inhibitors mediate their mobilizing effects are profoundly different: idelalisib (targets PI3Kδ) increased expression of S1PR1 and stimulated S1P-mediated migration, a process required for exit from lymph nodes; in contrast, fostamatinib (targets SYK) and ibrutinib (targets BTK) blocked chemotaxis toward chemokine, a process required for entry into lymph nodes and subsequent retention. This study therefore provides novel insight into the mobilizing effects of BCR signaling inhibitors, and, in particular, allows us to propose that the CLL cell–mobilizing effect of idelalisib results, at least in part, from reversal of BCR-mediated repression of S1PR1 expression on the malignant cells leading to enhanced egress from affected lymph nodes, whereas the mobilizing effect of ibrutinib is mediated by blockade of integrin-mediated signals required for tissue entry and retention. Our proposal is supported by clinical trial data demonstrating that the blood lymphocytosis observed in patients treated with idelalisib (39) reaches peak levels more rapidly than that induced by ibrutinib (40).

The BCR signaling pathway is central to the pathogenesis of CLL not only by providing prosurvival and proliferation signals (27, 28, 41), but also in maintaining malignant cell residency within lymph nodes (26, 42, 43). For example, BTK lies within the pathway controlling α_4_β_1_-mediated adhesion in BCR-stimulated cells (44) and can also mediate chemokine-induced migration and homing of normal B, mantle-cell lymphoma and CLL cells (26, 42, 45). Similarly, SYK is necessary for chemokine-induced migration and BCR-mediated adhesion of CLL cells (43, 46). Our demonstration that inhibition of SYK or BTK blocks the TEM of CLL cells toward chemokine is in agreement with these previous studies and supports the notion that the mobilizing effects of fostamatinib and ibrutinib result partly from enhanced exit from lymph nodes due to release of α_4_β_1_-dependent adhesive interactions and partly from reduced lymph node entry due to blockade of CCL21-directed TEM across the high endothelial venules.

In contrast to the effects seen with fostamatinib and ibrutinib, treatment of CLL cells with idelalisib had no effect on chemokine-induced migration in our experiments. Instead, we found that this compound strongly upregulated S1PR1 expression on CLL cells and significantly enhanced their migration toward S1P. Importantly, the increase in S1PR1 expression and function induced by idelalisib was still observed in the presence of accessory cells similar to those found in the microenvironmental niches where CLL cells reside in vivo. The in vivo relevance of our findings is further supported by evidence from normal B and T lymphocytes, suggesting that S1PR1-dependent egress overrides the proadhesive effects of Ag receptor engagement and chemokines. Thus, lymphocytes are retained in lymphoid and thymic tissues in the absence of functional S1PR1 expression (8, 47). Conversely, B cell responsiveness to chemokines in lymph nodes is reduced by enforced expression of S1PR1 (48). In summary, our results strongly suggest that idelalisib actively promotes S1P-directed egress by upregulating S1PR1.

Our study also provides insight into the mechanisms through which BCR signaling suppresses S1PR1 expression on CLL cells and, by extension, also on normal B cells. In particular, the differential effects of idelalisib and fostamatinib/ibrutinib on S1PR1 expression on CLL cells suggest that expression of the latter receptor is regulated by proximal BCR signals mediated by PI3Kδ but not distal BCR signals mediated by SYK and BTK. Our data also indicate that the regulation of S1PR1 by PI3K is isoform specific and mediated by the δ isoform specifically. These observations are in alignment with the specific role of PI3Kδ in transducing BCR-mediated signals that has been described elsewhere (49, 50), but do not illuminate the process any further. The mechanism through which active PI3Kδ regulates S1PR1 expression on lymphocytes is unclear. However, it could potentially involve guanine nucleotide-binding protein-coupled receptor kinase-2, which has been reported to desensitize S1PR1 (51).

Our results with idelalisib differ from those obtained in previous in vitro studies that have suggested that the compound is cytotoxic to CLL cells and blocks chemotaxis (20, 52). This discrepancy can be explained by the higher concentrations of idelalisib (5 and 10 μM, respectively) that were used in these other studies, with the potential for off-target effects. In contrast, the concentration of idelalisib selected for our study (1 μM) was the lowest required to block BCR-induced activation of Akt. It is also close to the peak plasma concentration of 2 μM predicted from a simplistic one-compartment model of drug disposition involving the administration of a standard dose of idelalisib (150 mg) to a patient of average size (75 kg) (34). Consequently, we believe that our observations with 1 μM idelalisib result exclusively from inhibition of PI3Kδ and are therefore more physiologically relevant than those resulting from higher drug concentrations.

Our findings with fostamatinib were only partially in keeping with those of Borge et al. (37). Thus, although the latter study showed that 1 μM fostamatinib had no effect on S1PR1 expression in CLL cells after 24-h culture, incubation with 5 μM fostamatinib resulted in increased S1PR1 expression. These findings need to be interpreted in the context of two important facts: first, our own findings (data not shown) and those of others (53) indicate that 1 μM fostamatinib can induce total blockade of BCR-induced signaling downstream of SYK (BTK and BLNK) in intact cells; second, the standard therapeutic dose of fostamatinib (500 mg) achieves a peak plasma concentration of 1.6 μM (21). Consequently, we believe that observations obtained with 1 μM fostamatinib are more physiologically relevant than those obtained with the 5 μM concentration.

By showing that the spontaneous upregulation of S1PR1 that was observed in a proportion of CLL cases could be reversed by BCR cross-linking and that idelalisib had the greatest effect on CLL cells that did not spontaneously upregulate S1PR1 in culture, the current study adds further weight to the growing idea that the BCR of CLL cells is chronically stimulated in vivo. The notion that CLL cells are subjected to ongoing in vivo antigenic stimulation is supported by studies of BCR glycosylation. Thus, the BCR expressed in CLL samples experiencing in vivo BCR engagement contains mannosyl residues consistent with receptor recycling (30). Our demonstration that the upregulation of S1PR1 by idelalisib is greater in unmutated CLL cells compared with mutated CLL cells is in keeping with the notion that unmutated CLL cells have greater in vivo BCR signaling activity (30). It also provides an explanation for the particular clinical benefit of idelalisib that is observed in unmutated CLL (54).

In summary, to our knowledge, our study is the first to show that BCR signaling represses S1PR1 expression and function on CLL cells, potentially leading to delayed egress from lymphoid tissues. To our knowledge, it is also the first study to suggest that different inhibitors of BCR signaling induce CLL cell mobilization through different mechanisms. In particular, by specifically blocking BCR-induced activation of PI3Kδ, we propose that idelalisib activity promotes S1P-mediated egress of CLL cells by relieving BCR-mediated repression of S1PR1 expression. Further work is now required to characterize the underlying mechanisms in the expectation that this may lead to the elucidation of new therapeutic targets.

## Supplementary Material

Data Supplement
