# Supplementary material for: Expression of Functional Sphingosine-1 Phosphate Receptor-1 Is Reduced by B Cell Receptor Signaling and Increased by Inhibition of PI3 Kinase δ but Not SYK or BTK in Chronic Lymphocytic Leukemia Cells
Source: J Immunol. 2015 Jan 28;194(5):2439–46. doi: 10.4049/jimmunol.1402304 (PMC4337486; doi:10.4049/jimmunol.1402304)
Supplement: Data Supplement [file JI_1402304.zip › JI_1402304_Supplemental_Material_1.pdf]

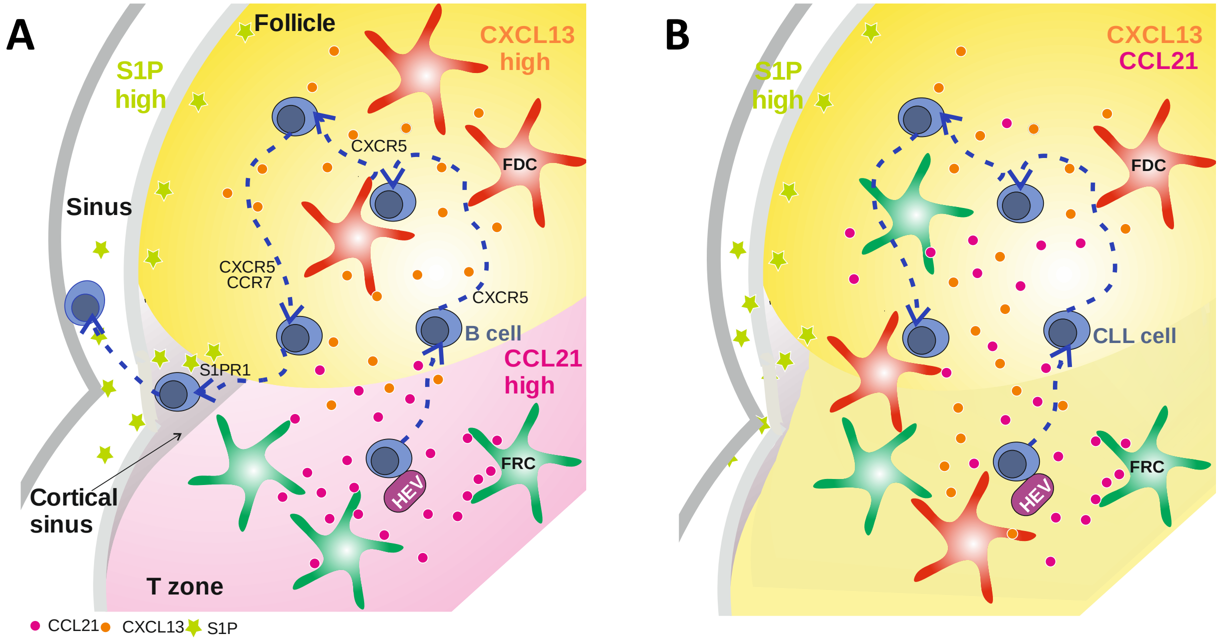

Supplementary Figure 1. Chemotactic signals involved in the tissue entry and egress of normal, and CLL, B cells. A. Normal lymph node. B lymphocytes enter the lymph nodes through the HEV in the T-cell zones in response to CCL21 and move along a gradient of CXCL13 towards the follicle in the search for antigen presented on the FDCs. The egress of normal B cells has not been studied, however it is dependent on S1PR1. It has been presumed that they follow a similar route to T cells, which in the absence of antigen encounter up-regulate CCR7 and S1PR1 and migrate back towards the T-cell zone along a gradient of CCL21, and finally exit the nodes through the cortical sinuses along a gradient of S1P. However, if T cells encounter antigen presented on the FDCs, transit time is increased to approximately 3 days due to down-regulation of S1PR1 and consequently an inability to migrate towards S1P. For ease, the route of travel is shown as linear path. However, in reality lymphocytes follow a more convoluted path and visit the follicular and interfollicular areas more than once during their passage through the node. **B.** CLL node. CLL cells also enter lymph nodes through the HEV in a CCR7/CCL21-dependent manner. However, the architecture within the node is totally effaced, and the malignant cells therefore populate the entire node. Both CCL21 and CXCL13 are found within the CLL node, and CLL cells have been shown to respond to both of these chemokines. Egress of CLL cells is delayed as compared with normal B cells but is not shown on this diagram since the mechanisms underlying this delay are not known.

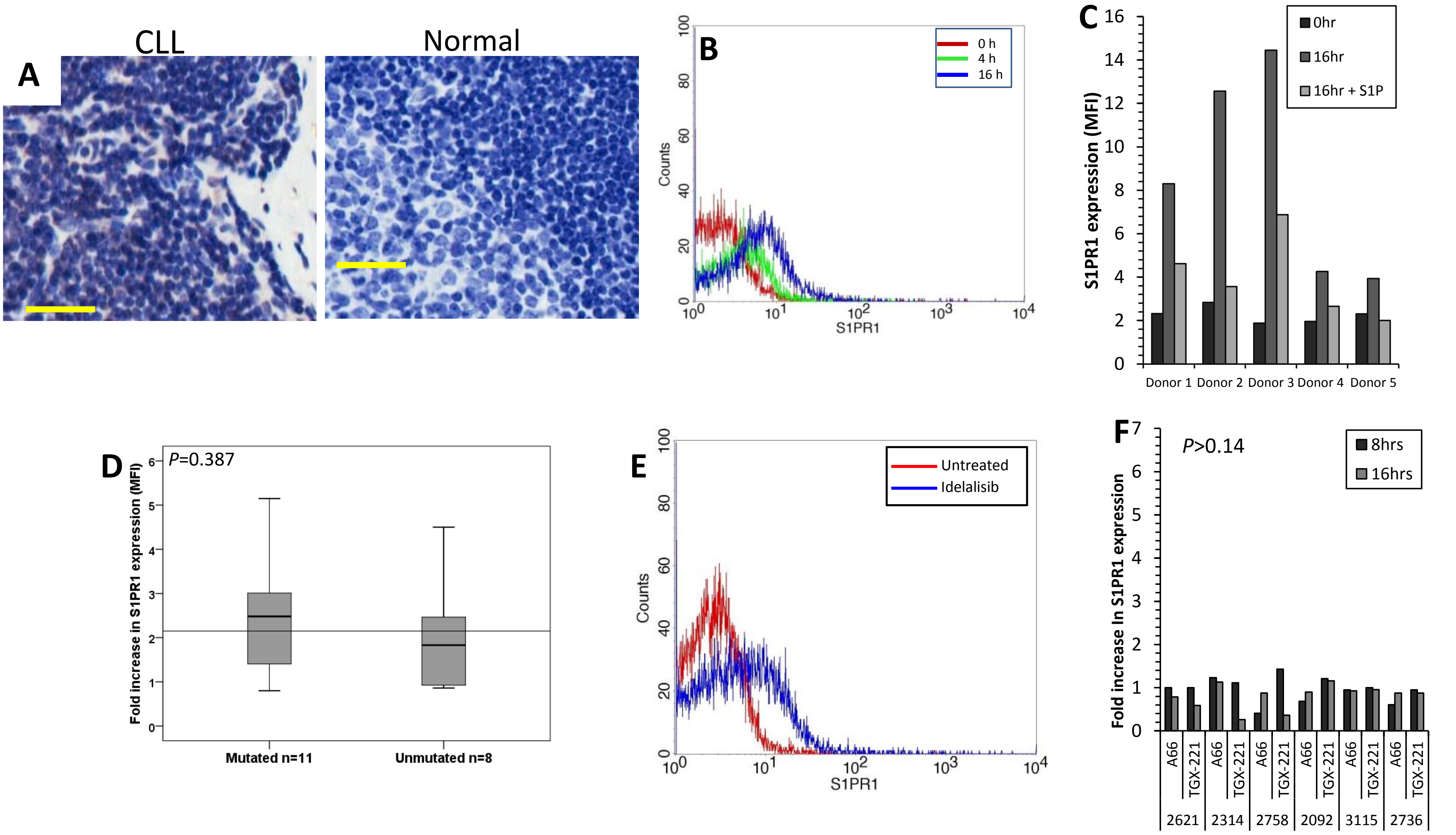

Supplementary Fig. 2 pt I

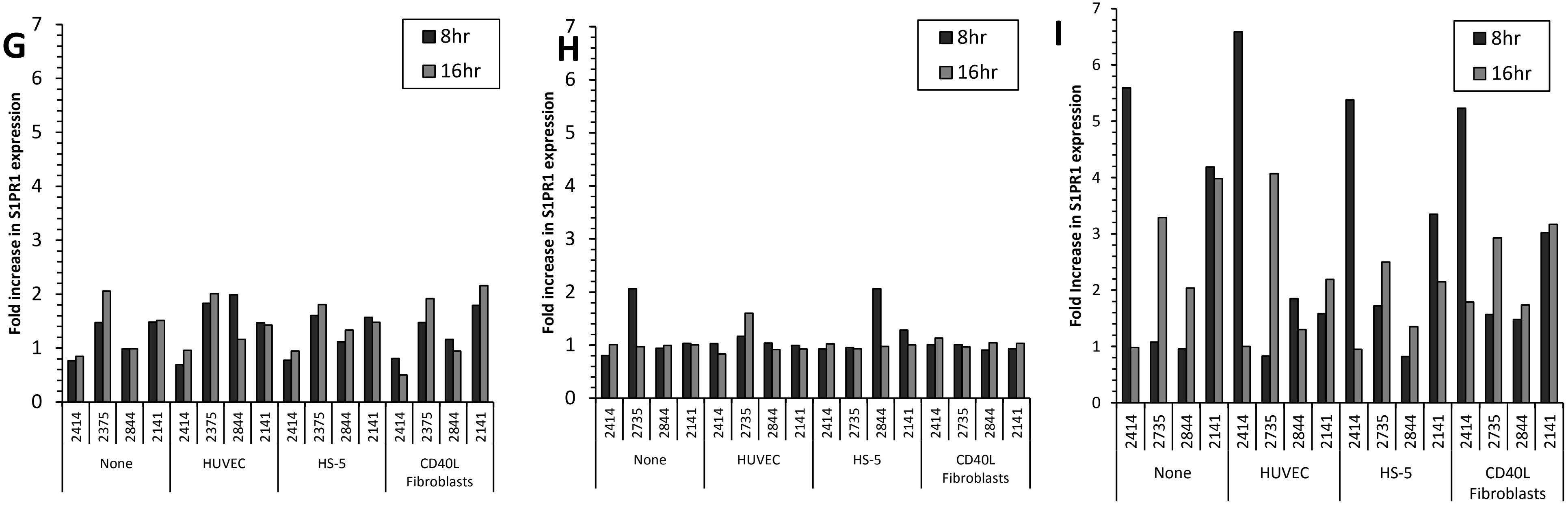

Supplementary Figure 2. Regulation of S1PR1 on normal and CLL B cells. A. Class-specific control staining for antibodies shown in Figure 1. Note no brown (DAB) stain is seen. Bar 50μM; original magnification x 20. **B.** Representative flow cytometry histogram showing an increase in S1PR1 expression on normal B cells after 4h and 16h culture in S1P-free medium. S1PR1 expression at 0 h was indistinguishable from that with the isotypic control antibody (not shown). **C.** B lymphocytes from 5 healthy donors were cultured in the presence or absence of S1P and examined for S1PR1 expression. The spontaneous increase in S1PR1 expression was abrogated by S1P. **D**. Box and whiskers plot relating fold increase in S1PR1 expression on CLL cells after 16 h in culture to IGHV mutational status. There was no significant difference in spontaneous S1PR up-regulation between the IGHV mutated versus un-mutated subgroups. Bar represents the grand median fold increase of both groups. **E.** CLL cells were cultured in the presence or absence of idelalisib and examined by flow cytometry for S1PR1 levels. A representative FACS plot (case 2758) shows up-regulation of S1PR1 by idelalisib at 16 h. S1PR1 expression in UT cells was indistinguishable from that with the isotypic control antibody (not shown). **F**. CLL cells from 6 patients were cultured in the presence or absence of 1 μM of inhibitors to PI3Kα (A66) or PI3Kβ (TGX-221) and examined by flow cytometry for S1PR1 levels. Neither inhibitor had any effect on S1PR1 levels (*P*>0.14). **G-I.** CLL cells from 4 patients were cultured on different stromal cell monolayers in the absence or presence of 1 μM of fostamatinib (**G**), ibrutinib (**H**) and idelalisib (**I**, see also Figure 3C) and examined by flow cytometry for S1PR1 expression. None of the stromal cell layers rendered CLL cells responsive to fostamatinib or ibrutinib, nor did they have any consistent effect on SP1R1 up-regulation by idelalisib.

Supplementary Figure 2 pt II

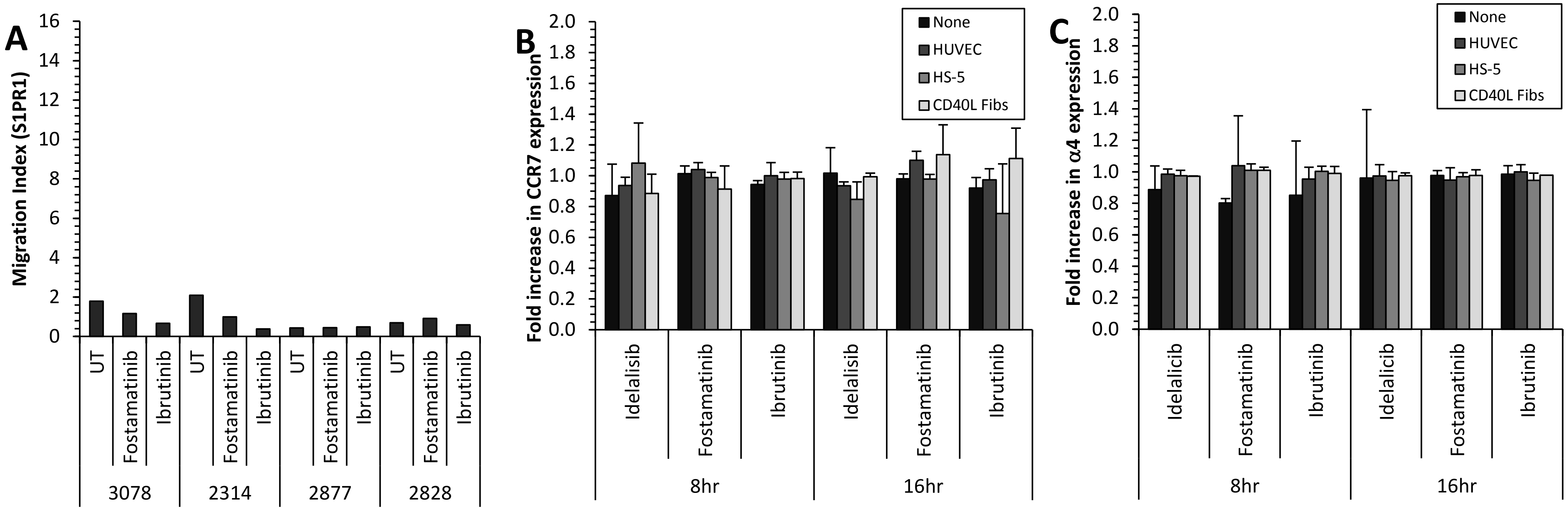

Supplementary Figure 3. Failure of fostamatinib and ibrutinib to induce CLL-cell migration towards S1P and failure of all 3 BCR signalling inhibitors to alter the expression of CCR7 and α4 integrin. A. CLL cells from 4 patients were cultured in the absence or presence of fostamatinib or ibrutinib (all at 1μM) and examined for migration towards S1P using HUVEC-coated transwells. In contrast to idelalisib (Figure 4A), fostamatinib and ibrutinib did not increase migration towards S1P. **B**. CLL cells from 4 patients were cultured on different stromal cell monolayers in the absence or presence of idelalisib, fostamatinib or ibrutinib (all at 1μM) and examined by flow cytometry for expression of CCR7. **C.** CLL cells from the same 4 patients were cultured on different stromal cell monolayers in the absence or presence of inhibitors and examined by flow cytometry for expression of or α4 integrin. None of the BCR signalling inhibitors had any effect on the expression of CCR7 α4 on any of the monolayers.

Patient	Stage at presentation Binet/Rai	Stage at study Binet/Rai	Nodes	WBC	V_H Mutation	Treatment	$\alpha4$	Fold increase in S1PR1	Idelalisib increase in S1PR1
2414	B/I	C/IV	Y	168	0.38	Pred, Chl	9.8	0.94	3.23
2375	B/II	B/II	Y	198.6	0	Y	36.9	2.16	2.46
2844	A/I	A/I	Y	71.6	11	NA	14.6	1.09	2.50
1866	C/IV	C/IV	Y	52	0	Chl/Pred	104.0	1.57	3.60
2621	NA	NA	Y	180.8		Y	36.1	4.42	5.30
2092	A/I	A/I	Y	82.4	4.5	N	6.9	2.92	3.80
2086	A/0	A/0	N	253	4.8	Chl/Pred	5.0	2.50	1.50
3115	A/0	A/0	N	62.8	0	CLB	23.0	2.77	3.60
2736	A/0	A/0	N	40.4	0	N	22.5	2.09	4.80
2877	A/0	A/0	N	160.8	8.5	NA	11.2	2.38	1.90
2314	A/0	B/II	Y	140	6.6	NA	NT	5.15	6.70
2758	B	B	Y	60.2	11.9	Chl/CamPred	NT	1.20	2.40
2120	NA	NA	NA	75.8	5.37	CHOP	NT	2.48	1.87
2581	A/I	B/II	Y	75	6.6	NA	NT	3.10	5.70
2752	A/0	A/0	N	75	4.51	NA	NT	3.50	1.58
3113	B	B	Y	174	0	NA	NT	0.86	3.50
3363	C	C	Y	15.9	0	FCR	NT	4.50	4.40
2141	A/0	A/0	N	60	6.19	NA	8.0	1.61	2.47
2877	A/0	A/0	N	160.8	8.5	NA	NT	0.80	6.72
2231	A/0	A/0	N	9.6	1.21	N	NT	1.00	1.85

Supplementary Table 1. Details of patients used Figure 1. CamPred-alemtuzimab plus methylprednisolone; Chl -chlorambusil; FCR – fludarabine, chorambucil, rituximab; CHOP - cyclophosphamide, doxorubicin, vincristine, prednisolone. Y – yes; N – no; NA - not available. NT - not tested.

Drug	Target	IC50 – cell free (nM)	IC50 – intact cells (μM)	Peak plasma concentration (μM)
Idelalisib	PI3Kδ	2.5	0.2-10	2
Ibrutinib	BTK	0.5	0.97-8.2	1.13
Fostamatinib	SYK	41	0.8-8.1	1.6

Supplementary Table 2. Concentrations of kinase inhibitors. IC50 values in cell free systems and intact cells (1-3). Peak plasma concentrations were derived using standard therapeutic doses. Note that the dose used in this study (1μM) is clinically achievable for all three drugs, and is within the IC50 range for intact cells.

1. Bodo J, Zhao X, Sharma A, Hill BT, Portell CA, Lannutti BJ, et al. The phosphatidylinositol 3-kinases (PI3K) inhibitor GS-1101 synergistically potentiates histone deacetylase inhibitor-induced proliferation inhibition and apoptosis through the inactivation of PI3K and extracellular signal-regulated kinase pathways. *British Journal of Haematology* 2013;163:72-80.
2. Chen L, Monti S, Juszczynski P, Daley J, Chen W, Witzig TE, et al. SYK-dependent tonic B-cell receptor signaling is a rational treatment target in diffuse large B-cell lymphoma. *Blood* 2008;111(4):2230-7.
3. Zheng X, Ding N, Song Y, Feng L, Zhu J. Different sensitivity of germinal center B cell-like diffuse large B cell lymphoma cells towards ibrutinib treatment. *Cancer Cell International* 2014;14(1):32.
